# Rutin Alleviates Zearalenone-Induced Endoplasmic Reticulum Stress and Mitochondrial Pathway Apoptosis in Porcine Endometrial Stromal Cells by Promoting the Expression of Nrf2

**DOI:** 10.3390/toxins17010007

**Published:** 2024-12-26

**Authors:** Chuangjiang Chen, Chenlong Wang, Hui Jiang, Mengya Wang, Sajid Ur Rahman, Changjiang Chen, Hongyan Ding, Chang Zhao, Wanyue Huang, Xichun Wang

**Affiliations:** 1 College of Veterinary Medicine, Anhui Agricultural University, Hefei 230036, China; 22720207@stu.ahau.edu.cn (C.C.); wangchenlong@stu.ahau.edu.cn (C.W.); jh1d32h@163.com (H.J.); 23720361@stu.ahau.edu.cn (M.W.); chang_zhao@ahau.edu.cn (C.Z.); huangwanyue@ahau.edu.cn (W.H.); 2 School of Public Health and Emergency Management, School of Medicine, Southern University of Science and Technology, Shenzhen 518055, China; sajid@sustech.edu.cn; 3 Huangyuan County Animal Husbandry and Veterinary Station, Huangyuan County Agriculture and Rural affairs Bureau, Xining 812100, China; 15352912913@163.com; 4 Anhui Province Key Laboratory of Livestock and Poultry Product Safety Engineering, Institute of Animal Science and Veterinary Medicine, Anhui Academy of Agricultural Sciences, Hefei 230001, China; dinghy1988@163.com

**Keywords:** zearalenone, rutin, Nrf2, endoplasmic reticulum stress, apoptosi

## Abstract

Zearalenone (ZEA) is a mycotoxin commonly found in moldy cereals and has a range of toxic effects that have seriously affected animal husbandry. Rutin, a natural flavonoid with antioxidant activities, has been studied for its potential involvement in mitigating ZEA-induced apoptosis in porcine endometrial stromal cells (ESCs) and its potential molecular mechanism, particularly concerning the expression of Nrf2. This study investigates the molecular pathways by which rutin alleviates ZEA-induced ESC apoptosis, focusing on the role of Nrf2. Experimental data reveal that ZEA suppresses Nrf2 nuclear translocation and reduces mitochondrial membrane potential (MMP), leading to oxidative stress, endoplasmic reticulum stress (ERS), and mitochondrial pathway-driven apoptosis. Notably, rutin mitigates ZEA-induced apoptosis through Nrf2 activation. These findings highlight Nrf2 as a critical factor in rutin’s protective effects against ZEA-induced apoptosis, offering valuable insights for the clinical prevention and treatment of ZEA toxicity.

## 1. Introduction

Zearalenone (ZEA) is a non-steroidal mycotoxin primarily produced by *Fusarium graminea* and commonly found in contaminated crops such as corn and wheat [1,2]. ZEA can bind with estrogen receptors, leading to disruptions in estrogen metabolism, alterations in the structure and function of reproductive organs, and adverse effects on the reproductive capacity of animals [3]. ZEA exposure can induce false estrus, miscarriage, premature birth, stillbirth, and other symptoms in female animals [4]. Furthermore, ZEA has been linked to decreased semen quality and interference with sperm production in male animals [5]. Different animal species exhibit varying sensitivity towards ZEA, with pigs being particularly vulnerable among economically significant livestock [6].

Endometrial stromal cells (ESCs) produce many cytokines that influence uterine epithelial function and play a critical role in female reproductive function, which undergo morphological and functional changes when exposed to toxins and are an important model for studying the molecular mechanism of endometrial cell response to toxin damage [7]. Previously, Yi and colleagues discovered that ZEA induces necrotic apoptosis in goat ESCs [8]. Our prior research indicates that ZEA induces mitochondrial fission, disrupts their ultrastructure, and promotes ESCs apoptosis [9]. Due to ZEA’s high thermal stability, there is currently no effective antidote, highlighting the need to identify protective agents against ZEA-induced ESCs damage.

Rutin, also known as vitamin P, is a naturally occurring flavonoid in common fruits and vegetables and grains such as grapes, apples, and buckwheat [10]. Rutin exerts many biological activities, such as antioxidant, anti-tumor, antibacterial, and anti-cancer [11]. Studies have shown that plant polyphenols, especially flavonoids, promote animal growth and development and have many biological activities, including antioxidant and immune regulation, which attracted the attention of researchers worldwide [12]. It has been shown that rutin can effectively alleviate equine herpesvirus-induced oxidative stress through the Nrf2/HO-1 signaling pathway [13]. Wang and his colleagues infected mice with ZEA and found that rutin reduced ZEA-induced liver inflammation and damage by regulating gut microbiota and improving intestinal barrier function [14]. However, whether rutin can alleviate ZEA-induced ESC damage remains to be investigated.

Mitochondria and the endoplasmic reticulum (ER) are essential cellular components in the body, each with distinct yet critical roles. Mitochondria, often called the cell’s powerhouses, are double-membraned organelles that facilitate aerobic respiration, making them indispensable for energy production. ER, a sprawling network of membranes, synthesizes and folds proteins destined for secretion [15,16]. Toxins can adversely affect mitochondria and ER. Apoptosis in the mitochondrial pathway is mainly induced by intracellular factors such as oxidative stress and Ca^2+^ accumulation, which can cause changes in the mitochondrial membrane permeability, reduce mitochondrial membrane potential (MMP), promote the release of pro-apoptotic substances into the cytoplasm, and then cause a series of cascade reactions [17]. ERS is a protective stress response of cells that alleviates body damage through the unfolded protein response (UPR) [18]. However, persistent or severe ERS can cause a range of damage to cells. ERS is mainly mediated by IRE1α, PERK, or ATF6. These three sensors can trigger apoptosis signals through different pathways [19]. Mitochondrial and caspase-mediated pathways are central to ZEA-induced cell apoptosis [20]. ZEA treatment disrupts the mitochondrial function and induces ERS, producing multiple apoptosis-inducing factors and ultimately resulting in cell apoptosis [21]. However, whether rutin can alleviate ZEA-induced ERS and mitochondrial pathway apoptosis still needs to be further investigated.

Nrf2 is a crucial intracellular antioxidant factor [22]. Under physiological conditions, Nrf2 binds to its inhibitor Keap1 and is inactivated in the cytoplasm [23]. Upon exposure to stimuli like ROS, Keap1 undergoes oxidation and conformational changes, resulting in the release and translocation of Nrf2. This activation promotes the expression of cellular antioxidant factors, enhancing cell protection [24]. Research has found that ZEA promotes ROS and malondialdehyde production by downregulating Nrf2 expression. This induction subsequently leads to cell apoptosis [25]. There is also evidence suggesting that rutin upregulates the expression of HO-1 and AOE through Nrf2 activation, thereby reducing ROS levels and mitigating oxidative damage [26].

Based on the above theory, we hypothesized that rutin can alleviate the oxidative stress of ESCs by promoting the expression of Nrf2, thus alleviating ZEA-induced ERS and mitochondrial pathway apoptosis. Therefore, we used porcine ESCs as an in vitro model and Nrf2 as the target protein to determine the alleviating effect of rutin on ZEA-induced cell damage and its potential molecular mechanism. Our findings on the protective mechanism of rutin may provide a theoretical foundation for developing strategies to prevent and treat ZEA-related conditions.

## 2. Results

### 2.1. Experimental Concentration of ZEA and Rutin on Porcine ESCs

**Figure 1:**
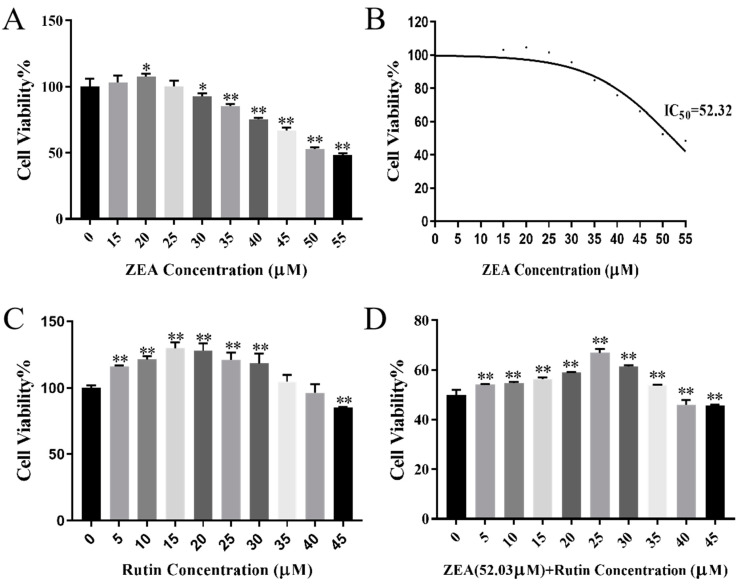
(**A**) Effect of different concentrations of ZEA on cell viability. (**B**) Prediction of the ZEA IC_50_ concentration by GraphPad. (**C**) Effect of different concentrations of rutin on cell viability. (**D**) Effects of different concentrations of rutin combined with ZEA on cell viability. Data presented above are means ± SD. * and ** indicate significant differences compared to the control group (*p* < 0.05 and *p* < 0.01).

As shown in Figure 1A, compared to the control group, ZEA exhibited a growth-promoting effect at concentrations below 20 μM. However, when the concentration was more than 30 μM, cell viability decreased with the increase in the ZEA concentration (*p* < 0.05). GraphPad predicted that the IC_50_ concentration of ZEA was 52.03 μM, so 52.03 μM ZEA was selected for subsequent experiments, as shown in Figure 1B. As shown in Figure 1C, with increasing the rutin concentration, cell survival initially increased and then decreased. When the concentration of rutin was 45 μM, cell viability decreased significantly (*p* < 0.01). Therefore, 0–45 μM was selected for subsequent concentration screening of the combined effects. As shown in Figure 1D, when the rutin concentration was 25 μM, the protective effect of the rutin concentration was adequate compared to the control group (with 52.32 μM ZEA added). Therefore, the concentration of rutin was 25 μM for subsequent experiments.

### 2.2. Rutin Promotes the Expression of Nrf2 in Porcine ESCs

According to the optimal interaction concentration of ZEA and rutin measured in Section 2.1, we set up the control group (CON), Z group (ZEA), R group (rutin), and Z+R group (ZEA + rutin) for follow-up tests.

**Figure 2:**
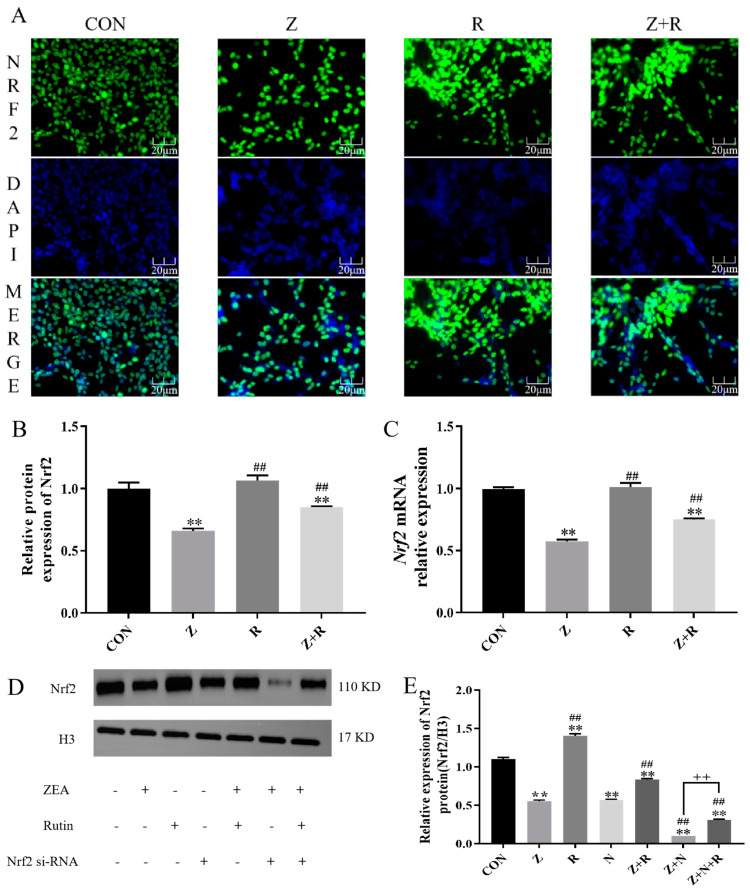
(**A**) The green fluorescence represents Nrf2, and the blue fluorescence represents the nucleus. (**B**) Relative luminance of Nrf2 protein fluorescence (**C**) Relative expression level of *Nrf2* mRNA. (**D**) Nrf2 protein expression. (**E**) Relative expression of Nrf2 protein. Data presented above are means ± SD. ** indicated significant difference compared to control group; ## indicated significant difference compared to the ZEA group; ++ indicated significant difference between the Z+N+R and Z+N group (*p <* 0.05, *p* < 0.01).

Figure 2 shows the nucleation of Nrf2 in different treatments. Compared to the control group, the nuclear translocation of Nrf2 was significantly inhibited in all groups except the rutin group (*p* < 0.01). In the Z+R group, nuclear translocation of Nrf2 was increased compared to the ZEA group (*p* < 0.01).

The level of *Nrf2* mRNA was detected by qRT-PCR. The level of *Nrf2* mRNA after ZEA treatment was significantly lower than the control group (*p <* 0.01). The level of *Nrf2* mRNA in the Z+R group was significantly increased than in the ZEA group (*p <* 0.01). Therefore, we chose Nrf2 as the target protein for follow-up experiments.

After selecting Nrf2 as the target protein, we added the N group (si-*Nrf2*), Z+N group (ZEA + si-*Nrf2*), and Z+N+R group (ZEA + rutin + si-*Nrf2*) and then detected the expression of Nrf2. As shown in Figure 2E, it is further demonstrated that rutin can promote Nrf2 expression.

### 2.3. Rutin Enhances Antioxidant Enzyme Activity and Reduces ZEA-Induced Oxidative Damage in Porcine ESCs

**Figure 3:**
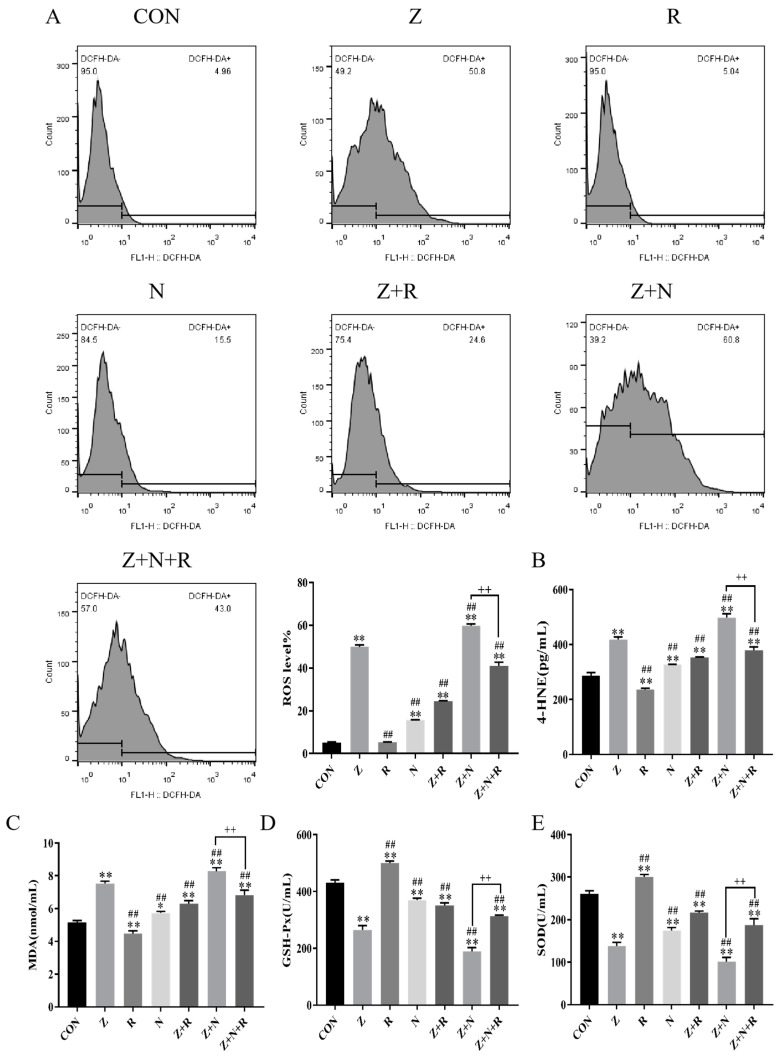
(**A**) ROS level detected by FCM and ROS horizontal bar chart. (**B**,**C**) The content of MDA and 4-HNE. (**D**,**E**) Activity of SOD and GSH-Px. Data presented above are means ± SD. ** indicated significant difference compared to the control group; ## indicated significant difference compared to the ZEA group; ++ indicated significant difference between the Z+N+R group and Z+N group (*p* < 0.05, *p* < 0.01).

In Figure 3A, compared to the control group, ROS levels were significantly higher in the ZEA group (*p* < 0.01). ROS levels in the Z+N group were further increased than in the ZEA group (*p* < 0.01). However, after rutin intervention, ROS levels were significantly reduced in both the Z+R and the Z+N+R groups (*p* < 0.01).

Figure 3B–E demonstrate that in the ZEA group, 4-HNE and MDA levels were significantly higher compared to the control group, while antioxidant enzyme activity was markedly lower (*p* < 0.01). Silencing *Nrf2* intensified ZEA’s damaging effects compared to the ZEA group (*p* < 0.01). However, rutin intervention led to significant decreases in 4-HNE and MDA levels, coupled with increased antioxidant enzyme activity (*p* < 0.01). Compared to the Z+N group, the Z+N+R group showed a significant reduction in cellular oxidative stress levels (*p* < 0.01).

### 2.4. Rutin Alleviates ZEA-Induced Mitochondrial Membrane Potential Reduction in ESCs

**Figure 4:**
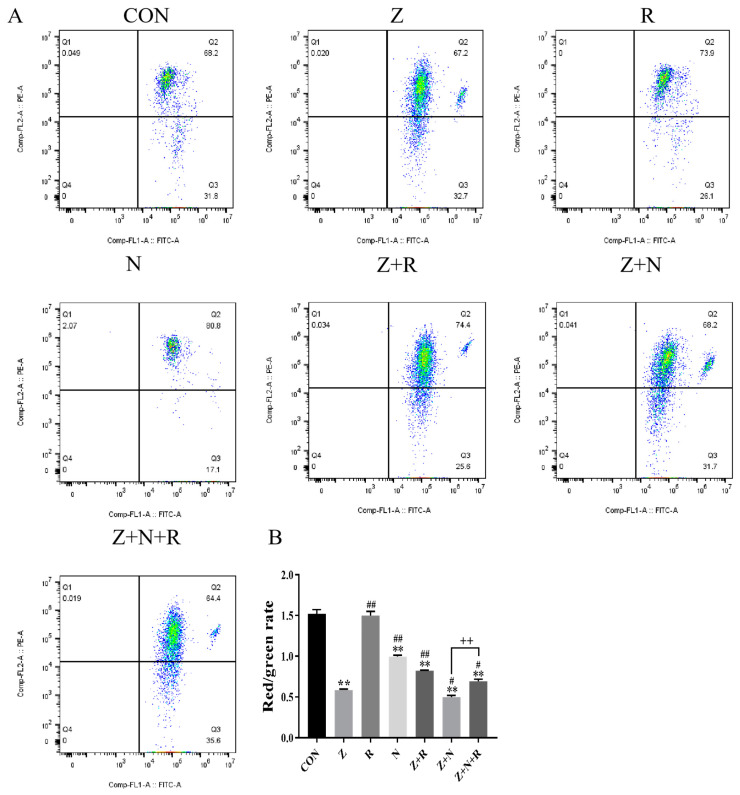
(**A**) The MMP detected by FCM (**B**) The histogram represents the MMP level. Data presented above are means ± SD. ** indicated significant difference compared to the control group; # and ## indicated significant difference compared to the ZEA group; ++ indicated significant difference between the Z+N+R and Z+N group (*p* < 0.05, *p* < 0.01).

In Figure 4B, the mitochondrial membrane potential in the ZEA group was significantly decreased compared to the control group (*p* < 0.01). The mitochondrial membrane potential in the Z+R group was increased than the ZEA group (*p <* 0.01), whereas it was further decreased in the Z+N group (*p <* 0.01). Notably, the mitochondrial membrane potential in the Z+N+R group was significantly higher than in the Z+N group (*p <* 0.01).

### 2.5. Rutin Alleviates ZEA-Induced Cell Apoptosis in Porcine ESCs

**Figure 5:**
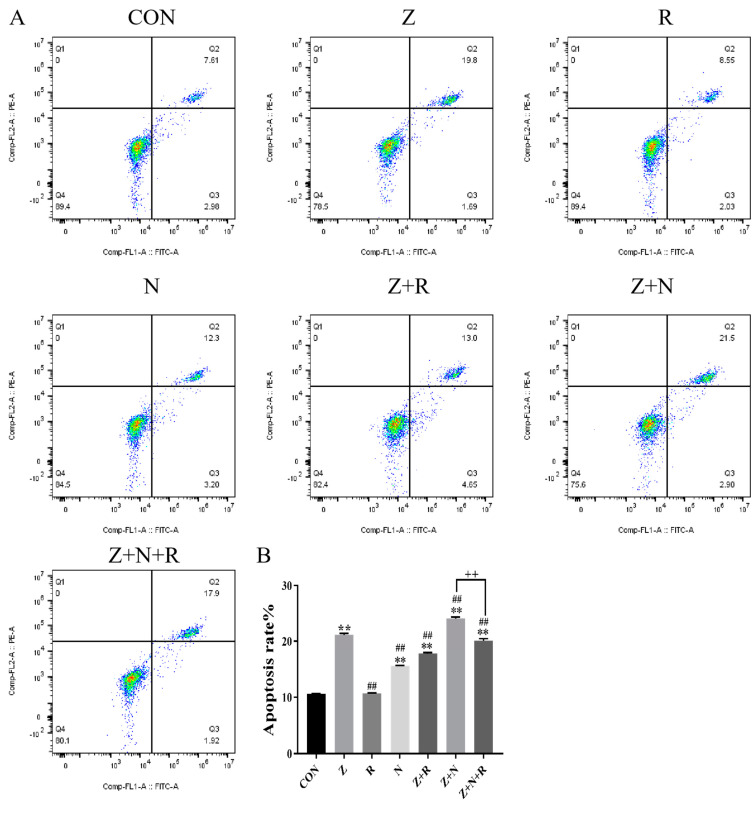
(**A**) The apoptosis rate detected by FCM (**B**) The histogram represents the apoptosis rate. Data presented above are means ± SD. ** indicated significant difference compared to the control group; ## indicated significant difference compared to the ZEA group; ++ indicated significant difference between the Z+N+R and Z+N group (*p* < 0.05, *p* < 0.01).

In Figure 5, compared to the control group, the apoptosis rate in the ZEA group was significantly increased (*p* < 0.01). Compared to the ZEA group, apoptosis was further increased after *Nrf2* silencing (*p* < 0.01). After the rutin intervention, the apoptosis rate of the Z+R and the Z+N+R group was significantly decreased (*p* < 0.01).

### 2.6. Effects of ZEA and Rutin on ESCs Gene Expression

**Figure 6:**
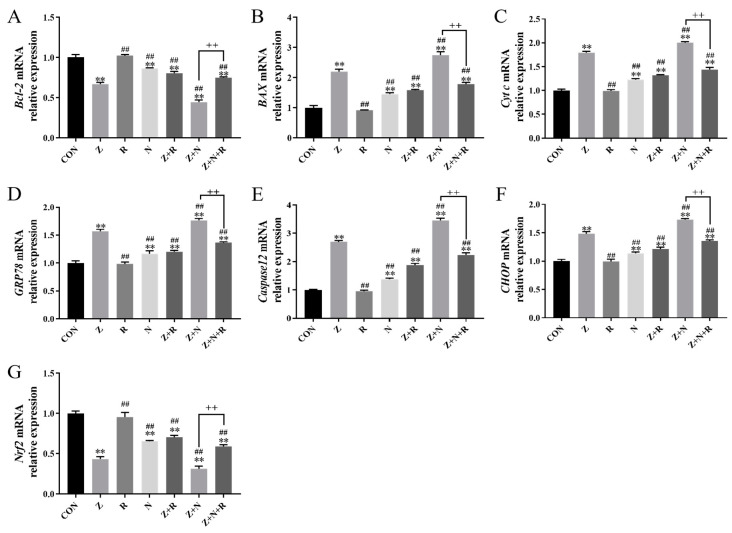
(**A**) *Bcl-2* mRNA expression. (**B**) *Bax* mRNA expression. (**C**) *Cyt c* mRNA expression. (**D**) *GRP78* mRNA expression. (**E**) *Caspase12* mRNA expression. (**F**) *CHOP* mRNA expression. (**G**) *Nrf2* mRNA expression. Data presented above are means ± SD. ** indicated significant difference compared to the control group; ## indicated significant difference compared to the ZEA group; ++ indicated significant difference between the Z+N+R and Z+N group (*p* < 0.05, *p* < 0.01).

Figure 6 shows the gene expression of cells in each group after treatment with different methods. ZEA significantly upregulated the expression of *Bax*, *Cyt c*, *GRP78*, *CHOP*, and *Caspase12* while downregulating the expression of *Bcl-2* and *Nrf2* compared to the control group (*p <* 0.01). Compared to the ZEA group, the expression of pro-apoptotic factors *Bax*, *Cyt c*, and ERS-related genes *GRP78*, *CHOP*, and *Caspase12* was further increased in the Z+N group, while the expression of *Bcl-2* and *Nrf2* was further decreased (*p <* 0.01). After rutin pretreatment, the expression of *Bax*, *Cyt c*, *GRP78*, *CHOP*, and *Caspase12* in the Z+R and Z+N+R groups was significantly decreased, while the expression of *Bcl-2* and *Nrf2* was significantly increased (*p <* 0.01).

### 2.7. Effects of ZEA and Rutin on ESCs Protein Expression

**Figure 7:**
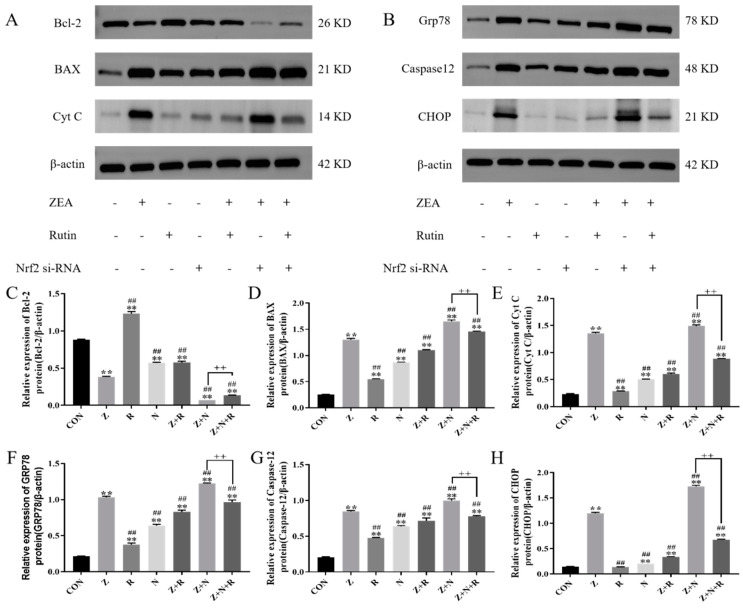
(**A**,**B**) Bcl-2, Bax, Cyt c, Grp78, Caspase12, and CHOP protein expression were detected by WB. (**C**–**H**) Relative expression of Bcl-2, Bax, Cyt C, Grp78, Caspase12, and CHOP protein. Data presented above are means ± SD. ** indicated significant difference compared to the control group; and ## indicated significant difference compared to the ZEA group; ++ indicated significant difference between the Z+N+R and Z+N group (*p* < 0.05, *p* < 0.01).

Figure 7 shows the expression of related proteins. Compared to the control group, the expression of Bax, Cyt c, GRP78, CHOP, and Caspase12 in the ZEA group was significantly increased, while the expression of Bcl-2 protein was significantly decreased (*p* < 0.01). Compared to the ZEA group, the expression of pro-apoptotic proteins Bax, Cyt c, and ERS-related proteins GRP78, CHOP, and Caspase12 in the Z+N group was significantly increased (*p* < 0.01). In contrast, the expression of anti-apoptotic proteins was significantly decreased (*p* < 0.01). However, after the rutin intervention, compared to both the ZEA and Z+N groups, the expression of anti-apoptotic proteins was significantly increased (*p <* 0.01), while the expression of Bax, Cyt C, GRP78, CHOP, and Caspase12 was significantly decreased, and the expression of Bcl-2 was significantly increased (*p <* 0.01).

## 3. Discussion

Mycotoxin pollution is very common in the world, which can hinder the healthy development of livestock relevant to agriculture and adversely impact the health and safety of both animals and humans [27]. ZEA is a heat-stable mycotoxin widely present in corn, wheat, and other crops. Its structure resembles endogenous estrogen, allowing it to competitively bind to estrogen receptors, disrupt estrogen metabolism, and induce reproductive dysfunction [3]. In addition, oxidative damage is an important mechanism by which ZEA exerts its toxic effects. Rutin is a natural estrogen compound from plants, which can promote the growth and development of animals and has various biological activities [12]. Studies have shown that most flavonoids can alleviate the toxic effects of ZEA. However, there are few reports on whether rutin can alleviate the toxic effects of ZEA, and the research on its potential molecular mechanism has not been reported. Nrf2 has been shown to enhance the body’s antioxidant capacity and alleviate oxidative stress and cytotoxicity. Therefore, ESCs were used as the model in vitro, and Nrf2 was used as the target gene to investigate the potential molecular mechanism of rutin alleviating the toxic damage of ZEA.

Oxidative stress refers to the imbalance of the redox state, which will produce a large number of ROS, causing further damage to the body [28]. It has been reported that ZEA induces an increase in ROS levels and promotes the apoptosis of ESCs [8]. Nrf2 is responsive to ROS and NO and, as one of the key antioxidant factors, plays a vital role in maintaining redox balance [29]. Studies have shown that ZEA can inhibit the expression of Nrf2 and thus stimulate the production of ROS [30]. We also found that ZEA inhibited the expression of Nrf2 and nuclear translocation, the enzyme activities of GSH-Px and SOD were decreased, while the contents of MDA and 4-HNE, as well as intracellular ROS levels, were significantly increased, which indicated that ZEA could inhibit the expression of Nrf2. Oxidative damage to ESCs is caused by inducing ROS and peroxide production.

Rutin is a flavonoid exhibiting antioxidant activity. Previous laboratory research suggests that supplementing rutin to the perinatal sheep diet can significantly lower the levels of MDA and H_2_O_2_ and enhance antioxidant enzyme activity. This action mitigates oxidative stress and apoptosis in the mammary glands of perinatal sheep by lowering the levels of downstream apoptotic markers [31]. In this study, the addition of rutin promoted the expression of Nrf2, increased the activity of antioxidant enzymes, and decreased the peroxide content and ROS levels, indicating that rutin effectively alleviated oxidative stress. However, when we silenced Nrf2, the relieving effect of rutin was inhibited. It indicates that rutin may reduce ZEA-induced cell damage by promoting Nrf2 expression and nuclear translocation, clearing ROS and peroxides, and inhibiting oxidative stress.

In mitochondria, the main source of ROS is the leakage of electrons during the respiration of electron chains, so mitochondria are vulnerable to ROS damage. When mitochondria are damaged, the MMP significantly decreases and changes mitochondrial permeability, which triggers the activation of the classical apoptotic cascade and eventually leads to irreversible apoptosis [32]. The Bax-like protein can form pores in the mitochondrial membrane, induce its permeability, and promote the release of Cyt c. On the other hand, Bcl-2-like proteins can inhibit this permeability [33]. In a classical apoptotic cascade, the Bax and Bcl-2 proteins undergo changes that promote the release of Cyt c. This is consistent with our findings. Mitochondria-dependent apoptosis is mainly regulated by Bcl-2 family proteins, and the release of Cyt c from mitochondria is also affected by the ratio of Bax to Bcl-2, which can promote the expression of Cyt c when its ratio increases [34]. During apoptosis, the ratio of Bax to Bcl-2 increases [35]. We found that ZEA reduced the level of the MMP and upregulated the ratio of Bax to Bcl-2 and the expression of Cyt c, indicating that ZEA induced ESCs apoptosis through the mitochondrial apoptotic pathway. After rutin intervention, the MMP level increased, the ratio of Bax to Bcl-2 and the expression of Cyt C were downregulated, which effectively alleviated the ZEA-induced mitochondrial apoptotic pathway. However, after Nrf2 was silenced, the relieving effect of rutin was significantly inhibited.

ROS and ERS are interrelated, with ROS acting as a signaling molecule in the UPR. This leads to the accumulation of intracellular ROS, which in turn activates ERS and triggers signaling pathways associated with apoptosis [36]. During ERS, PERK proteins are separated from GRP78, which is then activated through its own phosphorylation, promoting CHOP expression and initiating apoptosis [37]. CHOP is a vital gene involved in ERS-mediated apoptosis, and continuous activation of CHOP can activate the mitochondria-regulated apoptosis pathway through Bax [38]. We also found that ZEA treatment significantly upregulated the expression of Grp78 and CHOP and promoted the occurrence of apoptosis. Caspase12 is an ERS-induced apoptosis-specific factor that is not involved in the death receptor or mitochondrial apoptotic pathway [39]. We found that ZEA upregulates the expression of Caspase12 in ESCs, suggesting that ZEA promotes ESCs apoptosis by inducing ERS. When rutin was added, the expression of GRP78, CHOP, and Caspase12 was downregulated, alleviating ZEA-induced ERS. However, when we silenced the expression of Nrf2, the soothing effect of rutin was suppressed.

We believe that rutin can reduce ZEA-induced ESCs apoptosis by promoting the expression of Nrf2 and nuclear translocation, enhancing the body’s antioxidant capacity, alleviating oxidative stress, and inhibiting ERS and mitochondrial apoptotic pathway.

## 4. Conclusions

ZEA can disrupt the reproductive system of livestock, especially swine, and there is no effective antidote. The findings in the current study highlight the role of rutin in alleviating ZEA-induced cell damage, provide a new method for the detoxification of ZEA, and provide a theoretical basis for the clinical use of rutin. Nonetheless, certain limitations remain in this study. For instance, the in vivo absorption and bioavailability of rutin significantly influence its efficacy. Moving forward, we intend to investigate methods to enhance its utilization via in vivo experiments, thereby advancing the clinical application of rutin.

## 5. Materials and Methods

### 5.1. Chemicals and Reagents

ZEA (purity ≥ 99%) was purchased from the Academy of Agricultural Sciences (Shanghai, China). Rutin (purity ≥ 99%) standards were purchased from Hongye Biotechnology. (Shanghai, China). Nrf2 siRNA was purchased from Beijing Zixi Biotechnology. (Beijing, China). ESCs were purchased from Shanghai Qingqi Biotechnology. (Shanghai, China). South American FBS was sourced from EXCELL Biotechnology Co., Ltd. (Suzhou, China). DMEM High sugar base medium was purchased from Pricella (Wuhan, China). The ROS fluorescence test kit was purchased from Elabacience (Wuhan, China). The V-FITC/PI Kit was purchased from Unitech Biotechnology (Hangzhou, China). 4-hydroxynonenal (4-HNE), MDA, GSH-Px, and the T-SOD kit were bought from SINOBESTBIO (Shanghai, China). Antibodies to ACTIN, H3, Nrf2, BAX, GRP78, Caspase12, and Bcl-2 were purchased from Servicebio (Wuhan, China). Antibodies to Cyt c were purchased from BIOSS (Beijing, China). Antibodies to CHOP were purchased from Wuhan Sanying Biotechnology (Wuhan, China).

### 5.2. Cell Culture and Treatment

ESCs were cultured in a medium containing 10% FBS, 0.1 mg/mL streptomycin, 100 U/mL penicillin, and DMEM at 37 °C, and 5% CO_2_. Six point four grams of ZEA was dissolved in 1 mL DMSO, 2 mg rutin standard was dissolved in 16 μL DMSO, and 2.5 nmol Nrf2-siRNA was dissolved in 125 μL deionized water. The mother liquor stock solutions were stored at −20 °C and diluted to the used concentration with a complete medium before use.

When the cells grew to 60–70%, they were divided into different experimental groups, including the control group (CON), ZEA group (Z), rutin group (R), si-*Nrf2* group (N), ZEA + rutin group (Z+R), ZEA + si-*Nrf2* group (Z+N), and the ZEA + si-*Nrf2* + rutin group (Z+N+R). In the combined treatment group, ZEA, rutin, and siRNA were treated according to their respective concentrations. siNrf2 was pretreated 24 h in advance, and rutin was pretreated 2 h before ZEA exposure.

### 5.3. Analysis of Cell Viability

Cell viability was determined by the CCK-8 method. Cells (8 × 10^3^ cells/well) were inoculated on 96-well plates and cultured to a 50% density. Then, cells were treated according to the different methods described in Section 5.2. Next, 10 μL CCK-8 (5 mg/mL) was treated at 37 °C for 1 h, and absorbance was measured at 450 nm for cell viability assessment.

### 5.4. Transfection of Nrf2 siRNA

Specific *Nrf2* siRNA sequences (Forward primer: CAGUCUUCAUUGCUCCUAA; Reverse primer: UUAGGAGCAAUGAAGACUG) were used to inhibit the expression of Nrf2. Cells were seeded in 6-well plates at a density of 3 × 10^5^ cells per well and cultured to approximately 70% confluence. siRNA was then diluted in a transfection medium and transfected into the cells according to the manufacturer’s instructions. After 24 h of transfection, the cells were incubated in DMEM to prepare for subsequent treatments.

### 5.5. Detection of Apoptosis Rate

Cells were treated for 24 h and were washed with Hank’s balanced salt solution three times. The Fluo-3/Am working solution was incubated with cells at 37 °C for 0.5 h in the dark. Cells were collected by centrifugation, and apoptosis rate was analyzed using flow cytometry(FCM). The procedure has been described in detail earlier [40].

### 5.6. Detection of ROS Level

After 24 h of treatment, cells were incubated with DCFH-DA at 37 °C for 0.5 h in the dark. After centrifugation, cells were collected, and ROS levels were measured using FCM.

### 5.7. Determination of MMP

The cells were treated for 24 h, collected by centrifugation, washed with PBS, and re-suspended in JC-1 staining solution. They were then incubated at 37 °C with 5% CO_2_ for 20 min. After centrifugation at 600× *g* for 4 min, the cells were collected and washed with JC-1 staining buffer. Results were analyzed using FCM.

### 5.8. Determination of Oxidation and Antioxidant Levels

After cells were treated and cultured for 24 h, cells were digested by trypsin; the supernatant was collected and centrifuged at 3000 rpm for 10 min. MDA and 4-HNE levels and GSH-Px and T-SOD activities were detected according to the ELISA kit instructions.

### 5.9. Immunofluorescence

ESCs were seeded on sterile 24-well plates at a density of 8 × 10^4^ cells per well. Following treatment as described in Section 5.2, the cells were washed with PBS and then fixed with 4% paraformaldehyde for 0.5 h. Immunofluorescence staining was performed according to a previously published protocol [41].

### 5.10. Western Blotting

The cells (1.6 × 10^5^ cells/mL) were added into the holes of the sterile 6-well plate, treated according to Section 2.2, washed three times with PBS, scraped off the plate, and collected. Cells were lysed using RIPA lysis buffer, then centrifuged (15,000× *g*, 15 min, 4 °C), supernatant was collected, and the protein concentration was detected using the BCA kit. Details have been further described in a previous study [42].

### 5.11. Quantitative Real-Time Polymerase Chain Reaction Assay (qRT-PCR)

Total RNA was extracted from ESCs using Trizol reagent and reverse-transcribed into cDNA. β-actin acts as a housekeeping gene to normalize mRNA levels. Primer sequences in Table 1 were provided by Zhongkang Biotechnology Co., Ltd. (Hefei, China), and the specific operation procedure was referred to in our previous research report [24].

### 5.12. Statistical Analysis

The data are expressed as mean ± standard deviation (SD) and were analyzed using the t-test and One-way analysis of variance (ANOVA) (*n* = 3). SPSS 25.0 was used for statistical analysis, and GraphPad 9.5 Prism was used to create the bar charts. Results were considered statistically significant when *p* < 0.05.

## Figures and Tables

**Table 1:** qRT-PCR primer sequences.

Genes	Accession Number	Primer	(5′→3′) Sequences	Amplicon Size
*β-actin*	XM_021086047.1	F	GCCTACTGTGTGCTGAAGTTT	141
		R	GCTCTTCCCTTCTTCTCATTACC	
*Bax*	XM_003127290.5	F	TGGAGCAGGTGCCTCAGGAT	171
		R	TGCCGTCAGCAAACATTTCG	
*Bcl-2*	XM_021099593.1	F	GCCTATCTGGGCCATAAGTG	200
		R	TCCCTTTGGCAGTAAGTAGC	
*Caspase12*	NC_010451.4	F	GAGACAGCTCAAATTGCAGG	101
		R	TTCGCCTCTCTTTCTCCATC	
*CHOP*	NM_001144845.1	F	CTTCACCACTCTTGACCCTG	170
		R	CACTTTGTTTCCGTTTCCTG	
*Cytc*	XM_003127002.4	F	TACCTTTGTGTTAGGGCTAGAG	110
		R	TGTCTCTGTCAGCGTCAATAA	
*Grp78*	XM_001927795.7	F	GGCTCTACTCGCATCCCAAAG	115
		R	CCTGAACAGCAGCACCGTAA	
*Nrf2*	XM_013984303.2	F	ACTCAAGGGGTTGCGAAG	185
		R	GCAACTCAAACAGGGGAAG	

## Data Availability

The original contributions presented in this study are included in the article/Supplementary Material. Further inquiries can be directed to the corresponding author(s).

## Supplementary Materials

The following supporting information can be downloaded at: https://www.mdpi.com/article/10.3390/toxins17010007/s1, Figure S1: Chemical structural formulae of rutin. Reference [43] is cited in the supplementary materials.

## Abbreviations

Zearalenone (ZEA); endometrial stromal cells (ESCs); reticulum stress (ERS); Nuclear factor erythroid 2-related factor 2 (Nrf2); 4-hydroxynonenoic acid (4-HNE); Cytochrome C (Cyt c); Bcl2-Associated X (Bax); B-cell lymphoma-2 (Bcl2); glucose-regulated protein 78 (Grp78); C/EBP homologous protein (CHOP); cysteinyl aspartate specific proteinase 12 (Caspase12)
