# Supplementary material for: Rutin Alleviates Zearalenone-Induced Endoplasmic Reticulum Stress and Mitochondrial Pathway Apoptosis in Porcine Endometrial Stromal Cells by Promoting the Expression of Nrf2
Source: Toxins (Basel). 2024 Dec 26;17(1):7. doi: 10.3390/toxins17010007 (PMC11769520; doi:10.3390/toxins17010007)
Supplement: Supplementary file 1 [file toxins-17-00007-s001.zip › toxins-3355645-supplementary.pdf]

toxins

Supplementary Materials: Rutin Alleviates Zearalenone-Induced Endoplasmic Reticulum Stress and Mitochondrial Pathway Apoptosis in Porcine Endometrial Stromal Cells by Promoting the Expression of Nrf2

Chuangjiang Chen, Chenlong Wang, Hui Jiang, Mengya Wang, Sajid Ur Rahman, Changjiang Chen, Hongyan Ding, Chang Zhao, Wanyue Huang and Xichun Wang

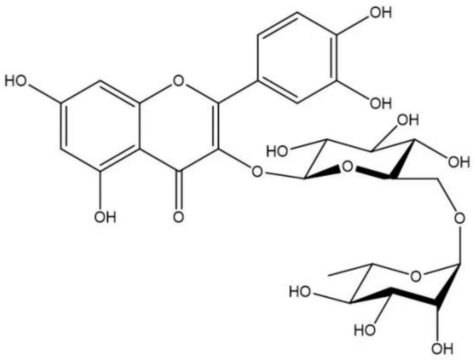

Figure S1. Chemical structural formulae of Rutin.

Rutin is a 3-part flavonol ligand composed of quercetin, glucose and rhamnose. It contains a benzo (γ) pyran ring structure, the A ring is a meso-diphenol structure, the B ring contains an o-diphenol structure, and the C ring is connected to a glucose group containing 3 hydroxyl groups and a rhamnose group containing 3 hydroxyl groups, i.e., rutin contains 4 phenolic hydroxyl groups and 6 glycosidic hydroxyl groups, which are more reactive and able to capture free radicals, which is the source of rutin's antioxidant activity.

Figure from the paper (Negm El-Dein, S., Hussein, A., Abu-Bakr, M.S. et al. Rutin and luteolin purified from Gerbera jamesonii and Chrysanthemum 'dante purple' flowers exert remarkable protection against the enteropathogen, E. coli O78, in poultry. Discov Food 4, 139 (2024). https://doi.org/10.1007/s44187-024-00217-1).
